# Inhibition of proanthocyanidin A2 on porcine reproductive and respiratory syndrome virus replication *in vitro*

**DOI:** 10.1371/journal.pone.0193309

**Published:** 2018-02-28

**Authors:** Mingxin Zhang, Qianqian Wu, Yao Chen, Mubing Duan, Ge Tian, Xianbo Deng, Yankuo Sun, Tong Zhou, Guihong Zhang, Weisan Chen, Jianxin Chen

**Affiliations:** 1 Guangdong Provincial Key Laboratory of Veterinary Pharmaceutics Development and Safety Evaluation, College of Veterinary Medicine, South China Agricultural University, Guangzhou, China; 2 Guangdong Provincial Key Laboratory of Prevention and Control for Severe Clinical Animal Diseases, College of Veterinary Medicine, South China Agricultural University, Guangzhou, China; 3 Department of Biochemistry and Genetics, La Trobe Institute for Molecular Science, La Trobe University, Melbourne, Victoria, Australia; Chinese University of Hong Kong, HONG KONG

## Abstract

Porcine reproductive and respiratory syndrome virus (PRRSV) is a widely prevalent and endemic swine pathogen that causes significant economic losses for the global pig industry annually. Currently, the most prevalent strategy for PRRSV control remains the prevention of virus transmission, with highly effective therapeutic agents and vaccines still lacking. Proanthocyanidin A2 (PA2) belongs to the family of tea polyphenols, which have been reported to exhibit a range of biological activities including anti-oxidative, cardio-protective, anti-tumoural, anti-bacterial, anti-viral, and anti-inflammatory effects *in vitro* as well as *in vivo*. Here, we demonstrate that PA2 exhibits potent anti-viral activity against PRRSV infection in Marc-145 cells. Similar inhibitory effects were also found in porcine alveolar macrophages, the primary target cell type of PRRSV infection in pigs *in vivo*. For traditional type II PRRSV CH-1a strain and high pathogenic GD-XH strain and GD-HD strain, PA2 exhibited broad-spectrum and comparable inhibitory activities *in vitro* with EC_50_ ranging from 2.2 to 3.2 μg/ml. Treatment of PRRSV-infected Marc-145 cells with PA2 significantly inhibited viral RNA synthesis, viral protein expression and progeny virus production in a dose-dependent manner. In addition, PA2 treatment reduced gene expressions of cytokines (TNF-α, IFN-α, IL-1β and IL-6) induced by PRRSV infection in PAMs. Mechanistically, PA2 inhibited PRRSV replication by targeting multiple pathways including blockade of viral entry and progeny virus release. Altogether, our findings suggest that PA2 has the potential to serve as a novel prophylactic and therapeutic strategies against PRRSV infection.

## Introduction

Porcine reproductive and respiratory syndrome (PRRS) is one of the most economically detrimental endemic diseases affecting pigs, and is characterized by severe reproductive failure in sows as well as pneumonia and an increased risk of secondary bacterial infections in piglets and growing pigs [1, 2]. The causative agent, porcine reproductive and respiratory syndrome virus (PRRSV), is a single-stranded positive-sense RNA virus belonging to the *Arteriviridae* family [3, 4]. The emergence of PRRSV as a pathogen was first documented in the 1980s as ‘mystery swine disease’, and is currently considered a global epizootic and endemic pathogen [5]. The virus has been classified into two major strains, the Type I (European) and Type II (North American) strains, which share only approximately 60% gene sequence homology [5, 6]. The genome of PRRSV is approximately 15 kb in size [7], and comprises a 50 base pair (bp) and 30 bp untranslated region (UTR) at the 5’ and 3’ ends respectively and 10 open reading frames (ORFs 1a, 1b, 2a, 2b, 3–7 and the newly identified ORF5a) [8–11]. ORFs 1a and 1b encode 12 non-structural proteins (NSP 1–12) involved in genome replication, transcription and the processing of viral polyproteins. Other ORFs encode structural proteins including four membrane glycoproteins, the matrix protein (M), and the nucleocapsid protein (N) [12–14]. Most importantly, PRRSV is reported to rapidly mutate at an estimated rate of 3.29×10^−3^ substitutions per nucleotide site per year and consequently evolves to form new strains frequently [15].

At present, vaccination remains the most prevalent method of controlling PRRSV infections. However, currently commercially available vaccines often fail to provide sufficient protection from infections due to the high degree of genomic variability found between different epidemic PRRSV strains [4]. Although modified live virus (MLV) vaccines confer increased protection against homologous virus strains compared to inactivated or recombinant vaccines and are thus predominantly used to control PRRS [15, 16], serious safety concerns remain as MLVs may regain virulence during serial passages in pigs [15, 17]. Consequently, a combined approach with pharmacological agents targeting PPRSV infections may be crucial for the effective management of PPRS.

Natural products and traditional remedies often harbor many bioactive compounds, which can provide an additional source of novel drug compounds apart from synthetic chemical libraries [18]. Such compounds have demonstrated anti-viral activities *in vitro* or *in vivo*, including those targeting PRRSV such as glycyrrhizin [19] flavaspidic acid AB [20], ouabain, valinomycin, bufalin [21], sodium tanshinone IIA sulfonate [22], morpholino oligomer [23] and hemin [24]. Despite this, no effective commercially available drugs currently exist for the treatment and management of PRRSV infections.

**Fig 1:**
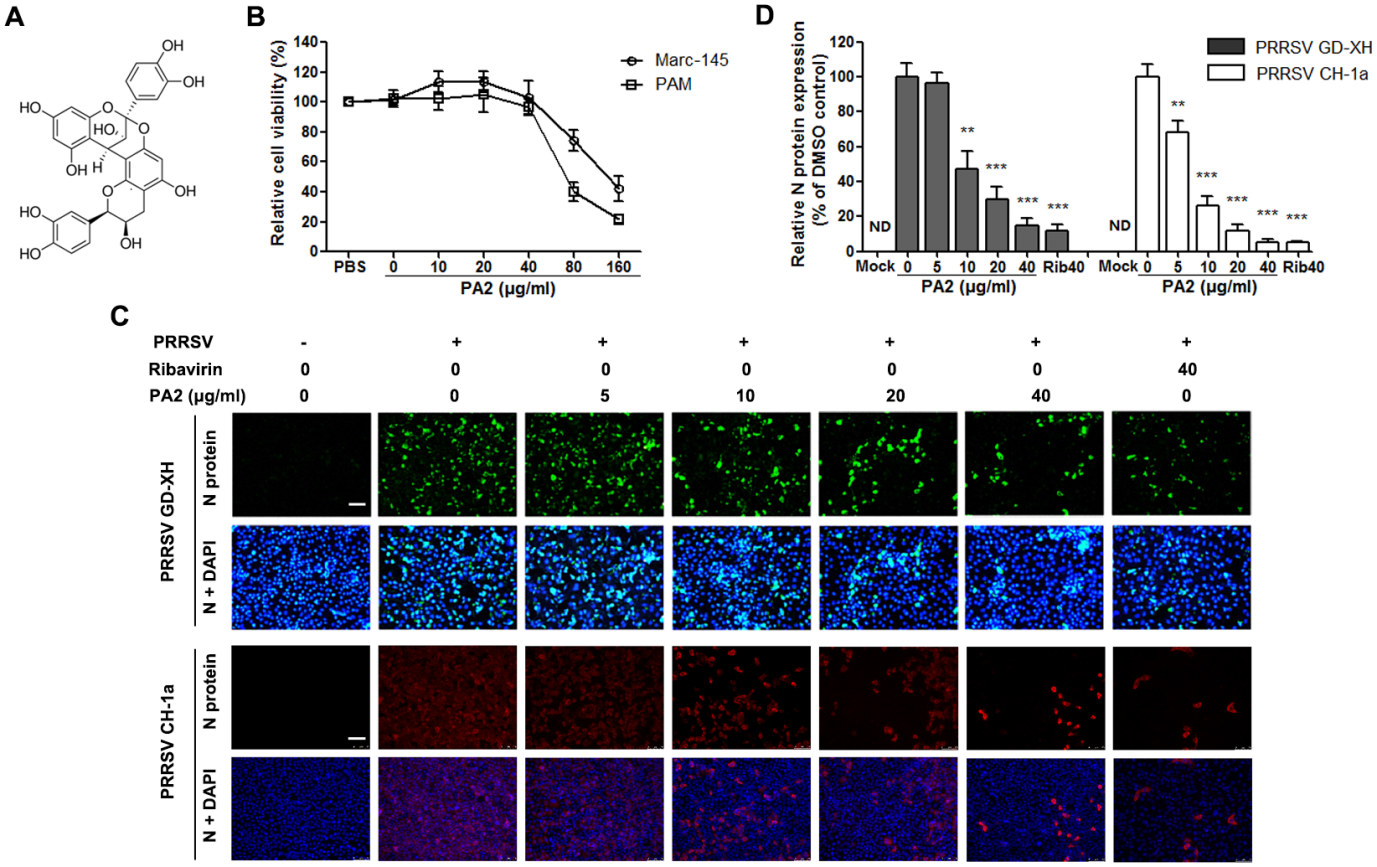
The anti-PRRSV activity and cellular toxicity of PA2 in Marc-145 cell cultures. (A) Chemical structures of proanthocyanidin A2 (PA2). (B) Cellular toxicity was examined in Marc-145 cells and PAMs using Cell Counting Kit-8 and was expressed as relative cell viability by comparing with the viable cells in the absence of compound (set up as 100%). (C and D) Antiviral activity of PA2 against the PRRSV CH-1a strain and high pathogenic GD-XH strain in Marc-145 cells was examined using IFA. Data shown in C are one representative of three independent experiments corresponding to D. Cells grown in 96-well plates were infected with PRRSV (0.05 MOI) for 2 h at 37°C and then cultured in fresh medium containing various concentrations of PA2. IFA for the N protein of PRRSV was performed at 48 hpi. Upper panels in C: GD-XH using Alexa Fluor 488-conjugated goat anti-mouse secondary antibody (green). Lower panels in C: CH-1a using Alexa Fluor 568-conjugated goat anti-mouse secondary antibody (red). Scale bar: 100 μm. Results shown in D are the mean values of relative fluorescence intensity of anti-N protein from three independent IFA experiments, and DMSO-treated control (0 μg/ml PA2) was set as 100%. Software Image J was used to analyze fluorescence intensity of IFA images. Statistical significances are denoted by *p < 0.05, **p < 0.01, and ***p < 0.001.

Proanthocyanidins are a group of naturally occurring polyphenolic bioflavonoids found in fruits, vegetables, nuts, seeds, particularly grape seeds, flowers and bark. Grape seed-extracted proanthocyanidin has been reported to exhibit a wide range of anti-oxidative, cardio-protective, anti-tumor, anti-bacterial, anti-viral, and anti-inflammatory activities *in vitro* [25–27]. Proanthocyanidin A2 (PA2) is a dimer of proanthocyanidine (Fig 1A) derived from the condensation of catechins [28]. PA2 and its analogues have been shown to exhibit anti-viral activities against herpes simplex virus (HSV), Coxsackie B virus (CBV) [29] and canine distemper virus (CDV) [30]. Based on its broad spectrum anti-viral effects, we postulated that PA2 may also exhibit anti-viral activity against PRRSV *in vitro*. In the present study, we demonstrate that PA2 significantly inhibited PRRSV infection in Marc-145 cells and porcine alveolar macrophages (PAMs) in a dose-dependent manner, through direct virus inactivation and the blockade of viral entry and progeny virus release. To our knowledge, this is the first report of the anti-PPRSV activities of PA2 *in vitro*.

## Materials and methods

### Cells, virus and chemicals

Marc-145 cells, a PRRSV-permissive cell line derived from MA-104 cells [31], were grown in Dulbecco’s minimum essential medium (DMEM, Gibco, USA) supplemented with 10% fetal bovine serum (FBS, Biological Industries, Israel), 100 IU/ml of penicillin and 100 μg/ml streptomycin at 37°C with 5% CO_2_.

Porcine alveolar macrophages (PAMs) were obtained from the lungs of 4- to 6-week-old PRRSV-negative Large-White piglets (Xinli Pig Farm, Wuzhou, China) by lung lavage according to a previously described method [32]. Briefly, the lungs and heart were removed, leaving a length of trachea in which to insert a suitable funnel, which was tied in place. The lungs were washed three times with pre-cooled phosphate buffered saline (PBS) solution containing penicillin (300 IU/ml) and streptomycin (300 μg/ml). Cells were centrifuged at 800 g for 10 minutes and resuspended in RPMI 1640 supplemented with 10% FBS and 100 IU/ml of penicillin and 100 μg/ml streptomycin at 1× 10^6^ cells/ml in 6-well plate, and then incubated at 37°C for 2 h. The suspending cells (mainly lymphocytes and red blood cells) were removed and adherent cells were PAMs [32] (shown in S1 Fig).

Three type II PRRSV strains including traditional CH-1a strain and highly pathogenic GD-XH strain and GD-HD strains [33] were propagated in Marc-145 cells in DMEM with 2% FBS (essential medium). Virus preparations were titrated and stored at -80°C. Virus titers were determined using a microtitration infectivity assay [34]. Briefly, virus preparations were 10-fold serially diluted in essential medium. Confluent monolayers of Marc-145 cells or PAMs prepared in 96-well plates were inoculated in quadruplicates with 100 μl of each sample and incubated for 2 h at 37°C. The inoculum was then discarded, and the cell monolayer replenished with fresh essential medium and incubated for an additional 72 h and monitored for cytopathic effects (CPE) daily. The titer of each preparation was calculated based on the amount of CPE and expressed as a 50% tissue culture infective dose (TCID_50_)/ml.

Proanthocyanidin A2 (PA2), isolated from *Lychee Seeds*, was a kind gift from Professor Guocai Wang, College of Pharmacy, Jinan University (Guangzhou, China), with a purity of ≥ 98% (HPLC). Mass spectrum of PA2 was shown in S2 Fig. Ribavirin, a broad-spectrum antiviral agent, was chosen as positive control and purchased form Star Lake Bioscience Co. Ltd (Zhaoqing, China) with a purity of ≥ 99%. All chemicals were dissolved in dimethyl sulfoxide (DMSO, Sigma, USA) and diluted with essential medium before use. The final concentration of DMSO in medium was less than 0.4%.

### Cell viability assay

The cytotoxicity of PA2 was evaluated using the Cell Counting Kit-8 (CCK8, DOJINDO, Japan). Confluent Marc-145 cells or PAMs were cultured in essential medium in 96-well plates containing 0, 10, 20, 40, 80 and 160 μg/ml PA2 for 72 h at 37°C. The medium was then removed and cells washed twice with PBS. CCK8 solution (10 μl) was added to each well and the plate incubated for an additional 2 h at 37°C. Cell viability was measured as the absorbance at 450 nm with a microplate reader (Thermo fisher scientific, USA) and expressed as a percentage of the control level. The mean optical density (OD) values from six wells per treatment were used as the cell viability index. The 50% cytotoxic concentration (CC_50_) was analyzed by GraphPad Prism 5.0 (GraphPad Software, San Diego, CA).

### Antiviral activity assay

The antiviral activity assay of compounds to be tested was performed to compare the *in vitro* inhibition of PRRSV replication. Marc-145 or PAM cell monolayers grown in 96-well plates were infected with PRRS virus (0.05 MOI for Marc-145 cells and 0.1 MOI for PAMs) and incubated in essential medium for 2 h at 37°C. Supernatants were removed and fresh DMEM containing different concentrations of each drug compound then added. Cells and supernatants were then collected at indicated time points post-infection and in total subjected to three freeze-thaw cycles at -80°C and 4°C respectively to ensure maximal release of cellular virions. Final supernatant viral titer was determined by the end point dilution assay using Marc-145 cells and expressed as log_10_ TCID_50_/ml [35]. The IC_50_ value (concentration of compound required to inhibit progeny viral titres by 50%) was determined by plotting the % inhibition of progeny viral titer as a function of compound concentration.

### Indirect immunofluorescence assay (IFA)

For immunostaining, PRRSV-infected or control cells were fixed with 4% paraformaldehyde for 15 min and then permeabilized with 0.2% Triton X-100 in PBS for 10 min at room temperature (RT). Cells were blocked with 5% bovine serum albumin (BSA) for 30 min at RT and then incubated with a mouse monoclonal antibody against the N-protein of PRRSV (clone 4A5, 1:400 dilution, MEDIAN Diagnostics, Korea) at 4°C overnight. After five washes with PBS, the cells were incubated for 1 h at RT with a goat anti-mouse secondary antibody conjugated with Alexa Fluor^®^ 488 (green) or Alexa Fluor^®^ 568 (red) (Cell Signaling Technology, USA) at 1:1000 dilution. Nuclei were counterstained using 4, 6-diamidino-2-phenylindole (DAPI: 1 μg/ml; Sigma Chemical Co., USA). Immunofluorescence was captured using the Leica DMI 4000B fluorescence microscope (Leica, Wetzlar, Germany).

### Real-time reverse transcriptase-PCR (RT-PCR)

Total RNA was extracted from cells or culture supernatants using the total RNA rapid extraction kit (Fastagen, Shanghai, China) following manufacturer’s instructions. RNA was reverse-transcribed into first-strand cDNA using a reverse transcription kit (TaKaRa, Japan). PCR amplification was performed on 1 μl of reverse-transcribed product with primers designed against PRRSV NSP9 and cytokines (TNF-α, IFN-α, IL-1β, IL-6) and specific primers for GAPDH (glyceraldehyde-3-phosphate dehydrogenase) used as the endogenous control. The primers used for PCR amplification are listed in Table 1. Real-time PCR was performed using 2×RealStar Green Power Mixture (containing SYBR Green I Dye) (Genstar, Beijing, China) on the CFX96 Real-time PCR system (Bio-Rad, USA). Relative mRNA expression were calculated by 2^-ΔΔCT^ method using DMSO-treated infected cells or DMSO-treated mock-infected cells as reference samples for determination of PRRSV NSP9 and cytokine gene, respectively[36, 37]. To assess the effect of PA2 on transcriptional activation of cytokines in PRRSV infected cells, the relative fold change of each cytokine gene was calculated and compared between virus-infected and mock-infected PAM cells and between PA2-treated virus-infected and virus-infected cells.

**Table 1:** Primer sequences for real-time PCR.

Name^a^	Sequences 5’ to 3’
NSP9-F	5’- CTAAGAGAGGTGGCCTGTCG -3’
NSP9-R	5’- GAGACTCGGCATACAGCACA -3’
GAPDH-F	5’- GCAAAGACTGAACCCACTAATTT -3’
GAPDH-R	5’- TTGCCTCTGTTGTTACTTGGAGAT -3’
TNF-α-F	5’- TGGTGGTGCCGACAGATGG-3’
TNF-α-R	5’- GGCTGATGGTGTGAGTGAGGAA-3’
IFN-α-F	5’- TCCAGCTCTTCAGCACAGAG-3’
IFN-α-R	5’- AGCTGCTGATCCAGTCCAGT-3’
IL-1β-F	5’- ACCTGGACCTTGGTTCTCTG-3’
IL-1β-R	5’- CATCTGCCTGATGCTCTTGT-3’
IL-6-F	5’- AATGTCGAGGCTGTGCAGATT-3’
IL-6-R	5’- TGGTGGCTTTGTCTGGATTC-3’

^a^F: forward primer; R: reverse primer

### Western blotting analysis

PRRSV-infected or control Marc-145 cells treated with PA2 were lysed in RIPA lysis buffer containing 1 mM phenylmethylsulfonylfluoride (Beyotime, Haimen, China) at 4°C. The supernatant was harvested after centrifugation (15,000 g for 30 min at 4°C) and the total protein for each sample measured with using the BCA protein assay kit (Beyotime, China). Ten micrograms of total protein per sample was electrophoresed onto a 12% SDS-PAGE gel and transferred to polyvinylidene-fluoride (PVDF) membranes (Millipore, USA). After blocking, membranes were incubated with a mouse anti-PRRSV N-protein monoclonal antibody (clone 4A5, MEDIAN Diagnostics, Korea) or a mouse anti-GAPDH monoclonal antibody (GoodHere, Hangzhou, China) at 1:1000 dilution at 4°C overnight. Anti-mouse IgG (H+L) (DyLight^®^ 800 Conjugate, 1:5000, CST, USA) was used as the secondary antibody for 1 h incubation at RT. The Odyssey system (LICOR, USA) was used to analyze the PVDF membranes.

### Virus binding assay

Marc-145 cells were incubated with DMEM containing PRRSV in the presence of PA2 or a control at 4°C for 2 h to facilitate virus binding. Cells were then washed three times with PBS to remove any unbound virus particles and chemicals and fresh medium then added. The cells were then cultured at 37°C for additional 46 h, and samples subsequently used for RT-PCR.

### Virus internalization assay

Marc-145 cells were incubated with essential medium containing PRRSV at 4°C for 2 h (a time-point which facilitates virus binding but not virus internalization). After three washes with PBS, cells were placed in fresh medium and cultured at 37°C to facilitate virus internalization. A serial dilutions of PA2 were then added at 0, 1, or 2 h and removed at 3 h post 37°C incubation. Cells were then washed three times with PBS to remove free virus particles and chemicals and incubated for additional 43 h at 37°C in fresh medium. The relative expression of viral mRNA level (expressed as a fold change) was analyzed by RT-PCR.

### Viral RNA replication assay

Marc-145 cells were infected with PRRSV for 6 h at 37°C, and then washed three times with PBS to remove free virus particles. Fresh medium containing 40 μg/ml PA2 was added and cells collected at 1, 2 or 3 h post PA2 addition for RT-PCR analysis.

### Virus release assay

Marc-145 cells were infected with PRRSV for 2 h at 37°C and then cultured in fresh medium for 24 h. The supernatants were then removed and the cells were cultured in fresh medium containing 40 μg/ml PA2. At 2 h post medium switching, the cell supernatants and cells were harvested separately for RT-PCR analysis.

### PA2 pretreatment of Marc-145 cells

To investigate whether PA2 inhibits PRRSV replication through altering host cell susceptibility, Marc-145 cells were pretreated with PA2 in essential medium for 2 h at 37°C. After three washes with PBS, cells were infected with PRRSV for 2 h and collected at 48 h post-infection (hpi) for RT-PCR analysis.

### Statistical analysis

All experiments were performed at least three times. Results were presented as mean ± standard deviation (SD). Statistical significance was determined by the Student’s *t* test using SPSS 17.0 (SPSS Inc., USA). Difference with *p* < 0.05 was considered to be statistically significant.

## Results

### PA2 inhibits PRRSV infection in Marc-145 cells

PA2 cytotoxicity was first measured using the Cell Counting Kit-8 on Marc-145 and PAM cells. As shown in Fig 1B, PA2 did not impair cell viability at even 40 μg/ml both in Marc-145 cells and PAMs. Interestingly, PA2, at 10 or 20 μg/ml, slightly increased Marc-145 cell viability but not PAMs viability. However, at concentrations of 80 and 160 μg/ml, PA2 exhibited significant and dose-dependent cytotoxic effects on both Marc-145 cells and PAMs compared to the PBS-treated control. The 50% cytotoxic concentration (CC_50_) of PA2 on Marc-145 cells and PAMs were 126.5 and 63.9 μg/ml, respectively.

Next, we investigated the effects of PA2 in Marc-145 cells infected with a traditional PRRSV strain CH-1a and a highly pathogenic PRRSV strain GD-XH, respectively, using indirect immunofluorescence assay (IFA). As shown in Fig 1C and 1D, PA2 significantly reduced PRRSV replication for both strains as reflected by a dose-dependent decrease in PPRSV N-protein staining levels. 40 μg/ml of PA2 exhibited similar inhibitions on both investigated PRRSV strains to that of 40 μg/ml of ribavirin, a well-known inhibitor of viral RNA polymerase used as a positive antiviral drug control.

**Fig 2:**
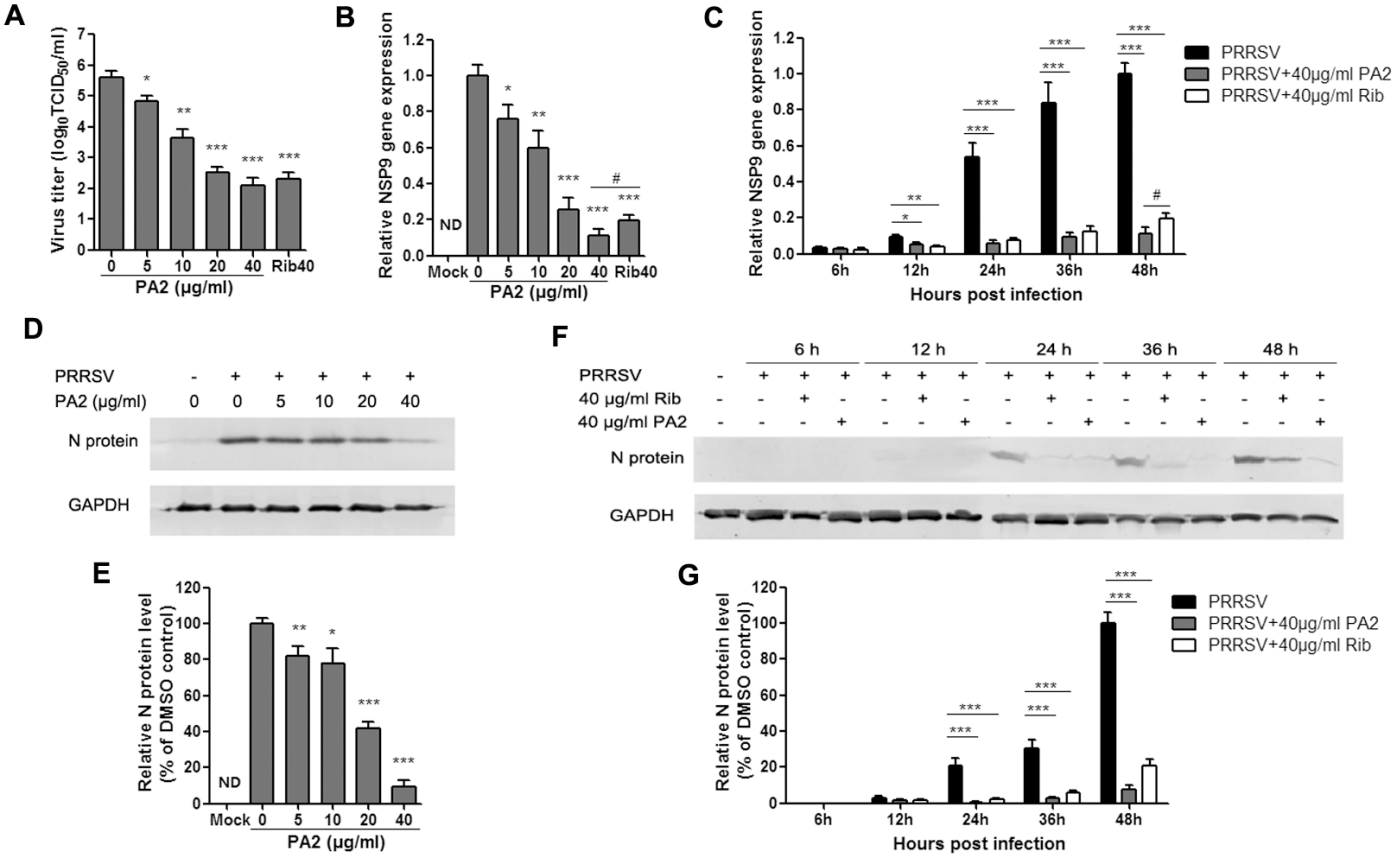
Confirmation of anti-PRRSV activity of PA2 in Marc-145 cell cultures. Cells grown in 6-well plates were infected with PRRSV GD-XH (0.05 MOI) for 2 h at 37°C and then cultured in fresh medium containing various concentrations of PA2. At 48 h (A, B, D and E) or indicated time-points (C, F and G) post infection, the samples were subjected to viral titer titration, or RT-PCR or Western blotting analysis. (A) The PRRSV titer was determined after treatment with PA2 for 48 h using the end point dilution assay and expressed as log_10_ TCID_50_/ml. (B and C) Relative PRRSV NSP9 mRNA level was analyzed using real-time RT-PCR at 48 h (B) or indicated time-points (C) after treatment with PA2. Expression of GAPDH was shown as the internal loading control, and DMSO-treated sample (0 μg/ml PA2) at 48 h was used as the reference control (set as 1). Results shown in A, B and C are the mean values from three independent experiments, and error bars represent standard deviations. (D and E) Expression of viral N protein in cells treated with various concentrations of PA2 for 48 h was detected by Western blotting. (F and G) Expression of viral N protein in cells treated with 40 μg/ml PA2 or 40 μg/ml ribavirin for various hours was detected by Western blotting. β-actin was used as a loading control. Results shown in E and G are normalized N protein levels based on the optical densities (OD) of bands from three independent experiments. Software Image J was used to analyze band OD; Results from PA2 treated samples were compared to those from DMSO-treated control groups (0 μg/ml PA2) for 48 h. Data shown in D and F are one representative of three independent experiments corresponding to E and G, respectively. Statistical significances are denoted by *p or ^#^p < 0.05, **p < 0.01, and ***p < 0.001.

To confirm the antiviral activity of PA2 against PRRSV, we examined the antiviral effects of different PA2 concentration on the GD-XH strain using virus titration, RT-PCR and Western-blotting assays at 48 hpi. As shown in Fig 2A, treatment with PA2 resulted in a notably significant reduction of PRRSV titer in a dose-dependent manner. Treatment with 40 μg/ml of PA2 produced a 2,000-fold reduction in progeny virus production compared to DMSO-treated control (0 μg/ml of PA2) (Fig 2A). The 50% inhibitory concentration (IC_50_) of PA2 against PRRSV GD-XH and CH-1a infection in Marc-145 cells were calculated to be 2.5 and 3.2 μg/ml, respectively, which are much lower than its CC_50_ values. Fig 2B shows that PA2 treatment at concentrations from 5 to 40 μg/ml significantly inhibited PRRSV NSP9 RNA levels in a dose-dependent manner compared with control DMSO-treated Marc-145 cells. A statistically significant increase in PRRSV inhibition was also observed using PA2 compared to ribavirin at 40 μg/ml (Fig 2B). PA2 treatment also significantly inhibited PRRSV N protein levels in a dose-dependent manner at concentrations from 5 to 40 μg/ml (Fig 2D and 2E).

We further studied the PRRSV inhibition kinetics of PA2 at 40 μg/ml. PRRSV-infected DMSO-treated control showed negligible viral RNA levels at 6 hpi, indicating that relatively few progeny viruses were produced during first 6 h. From 12 to 48 hpi, a continuous increase in viral RNA levels was detected (Fig 2C). Virus titer exhibited a similar profile with the mRNA levels at different time-points, as shown in S3 Fig. The addition of 40 μg/ml of PA2 significantly inhibited viral RNA levels at all time-points tested (Fig 2C). A similar pattern in viral N protein expression levels was also observed by Western blot following PA2 treatment (Fig 2F and 2G).

### PA2 inhibits PRRSV infection in PAMs

**Fig 3:**
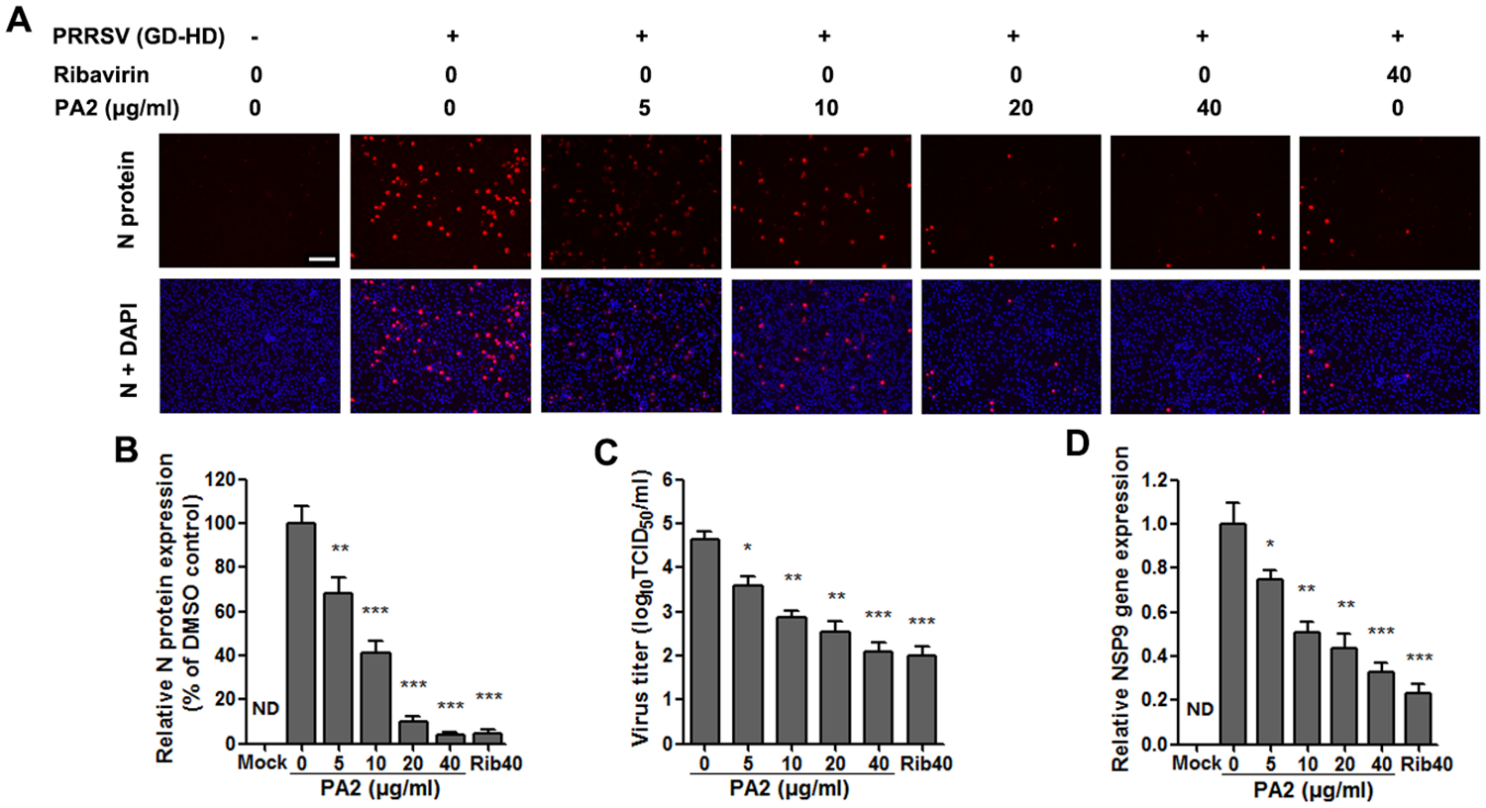
The anti-PRRSV activity of PA2 in PAM cultures. PAMs grown in 96-well plates (A and B) or 6-well plates (C and D) were infected with PRRSV GD-HD (0.1 MOI) for 2 h at 37°C and then cultured in fresh medium containing various concentrations of PA2. After treatment with PA2 for 24 h, the samples were subjected to IFA, or viral titer titration and RT-PCR analysis. (A and B) Indirect immunofluorescence assay for the N protein of PRRSV was performed at 24 hpi. For staining, Alexa Fluor 568-conjugated goat anti-mouse antibody was used as the secondary antibody (red), and nuclei were stained with DAPI (blue). Scale bar: 100 μm. Results shown in B are the mean values of relative fluorescence intensity of anti-N protein from three independent IFA experiments (DMSO-treated control as 100%), and A is one representative data from B. (C) The PRRSV titer was determined after treatment with PA2 for 24 h using the end point dilution assay and expressed as log_10_ TCID_50_/ml. (D) Relative PRRSV NSP9 mRNA expression of PA2 treated groups to DMSO-treated control (0 μg/ml PA2) (set as 1) was analyzed using real-time RT-PCR at 24 h after treatment with PA2. Results shown in C and D are the mean values from three independent experiments. *p < 0.05, **p < 0.01, and ***p < 0.001 compared to DMSO-treated control.

To investigate whether PA2 has anti-PRRSV activity in PAMs, which are the major target cells in infected pigs, we examined the antiviral effects of PA2 on the GD-HD strain in PAMs using IFA, virus titration and RT-PCR assays at 24 hpi. As shown in Fig 3A and 3B, PA2 significantly reduced PRRSV N-protein levels, reflecting dose-dependent PRRSV suppression. A notably significant reduction of PRRSV titer in a dose-dependent manner was also observed when PRRSV-infected PAMs were treated with PA2 (Fig 3C). Treatment with 40 μg/ml of PA2 produced a 600-fold reduction in progeny virus production compared to DMSO control (Fig 3C). The IC_50_ of PA2 against PRRSV GD-HD in PAMs was calculated to be 2.2 μg/ml, a similar PA2 concentration against PRRSV GD-XH in Marc-145 cells. A similar pattern in relative viral mRNA levels was also confirmed by RT-PCR following PA2 treatment, as shown in Fig 3D. Inhibitions of PA2 on PRRSV CH-1a and GD-XH infections in PAMs were also observed by RT-PCR analysis (shown in S4 Fig).

### PA2 blocks cellular entry of PRRSV

**Fig 4:**
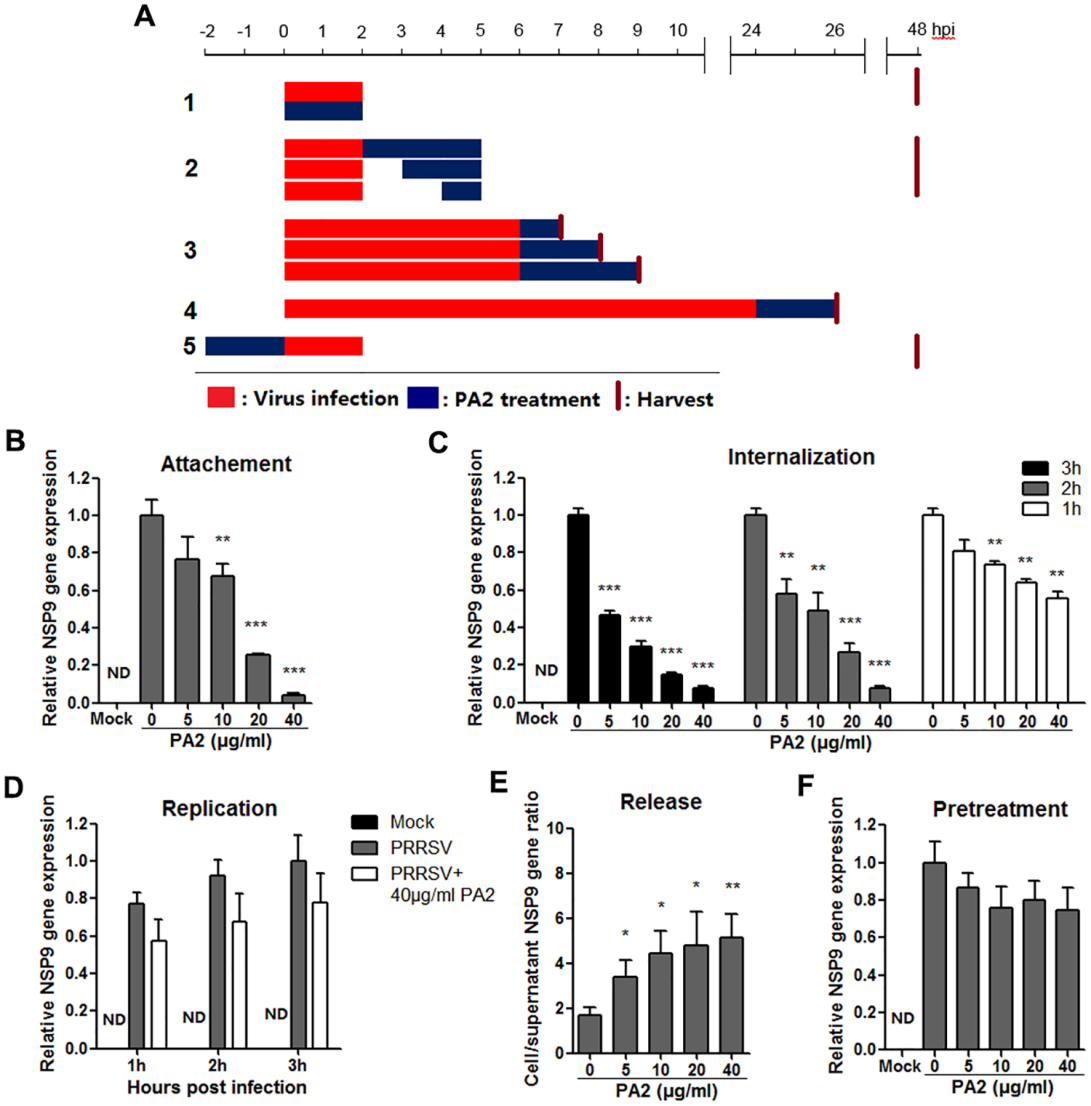
Effect of PA2 treatment on PRRSV entry, replication and release. Marc-145 cells were infected with the PRRSV GD-XH strain at 0.05 MOI. The infected cells were cultured in the presence of a series of concentrations of PA2 and collected at indicated time-points post infection for determination of the relative expression level of viral NSP9 mRNA by RT-PCR. Cellular GAPDH mRNA was used as the internal loading control and DMSO-treated sample (set as 1) was used as the reference control. (A) Different PA2 treatment schemes. Red bars represent PRRSV infection period, blue bars represent PA2 treatment period, and red vertical bars represent end of treatments and cell harvesting. (B) Viral binding was investigated by treatment A1; (C) Viral internalization was investigated by treatment A2; (D) Viral replication by treatment A3; (E) Viral release by treatment A4; and (F) PA2 pretreatment was performed by treatment A5. The data (in B, C, D, E and F) represent the mean ± standard deviation from three independent experiments. Statistical significances are denoted by *p < 0.05, **p < 0.01, and ***p < 0.001.

In order to dissect the *in vitro* mechanism of PA2 mediated PRRSV virus inhibition, we first examined the effects of PA2 on virus entry through cell attachment and subsequent internalization pathways. Marc-145 cells were incubated in the essential medium containing PRRSV in the presence or absence of PA2 at 4°C which facilitated virus binding but not cellular internalization (Fig 4A, treatment 1). Subsequent virus replication levels were significantly inhibited in a dose-dependent manner, suggesting that PA2 directly exerted inhibitory effects on PRRSV binding to Marc-145 cells (Fig 4B).

Kreutz and Nauwynck have previously reported that PRRSV is internalized from the surface of Marc-145 cells within 3 to 6 hpi [38]. Thus, to examine whether PA2 may also affect the internalization of PRRSV, we studied the effects of PA2 addition on PRRSV infected Marc-145 cells at 0, 1 or 2 hpi (Fig 4A, treatment 2). As shown in Fig 4C, virus replication was significantly inhibited in a dose-dependent manner when PA2 was added at 0 hpi to 2 hpi, suggesting that PA2 may also inhibit PRRSV internalization.

To confirm inhibitory effects of PA2 on attachment and internalization of PRRSV, a higher dose of PRRSV GD-XH (0.5 MOI) was used to infect Marc-145 cells. For attachment assay, PA2 was added together with virus to cells and incubated for 2 h at 4°C. For internalization assay, PA2 was added at 2 hpi and incubated for 3 h at 37°C. Then cells were collected and submitted to RT-PCR analysis. As shown in S5 Fig, PA2 blocked attachment and internalization of PRRSV in a dose-dependent manner.

### PA2 does not inhibit replication of viral RNA but blocks the release of progeny virus particles

Previous studies have demonstrated that PRRSV progeny viruses are released by 8 hpi [19, 38]. To explore whether PA2 directly inhibits the replication of viral RNA, Marc-145 cells were incubated with PRRSV for 6 h at 37°C and then cultured in fresh medium containing 40 μg/ml of PA2. Cells were collected at 7, 8, and 9 hpi and used for RT-PCR analysis (Fig 4A, treatment 3). PA2 treatment did not significantly reduce the expression levels of virus RNA compared to that in control treated cells, suggesting that PA2 does not affect PRRSV RNA replication (Fig 4D).

We wondered whether PA2 could also affect the later as well as earlier stages of the PRRSV life cycle. To investigate these, Marc-145 cells were infected by PRRSV for 24 h at 37°C and the cells were then cultured in fresh medium containing varying concentrations of PA2 for another 2 h. Subsequently, the cells and supernatants were separately collected for the quantification of NSP9 RNA by RT-PCR (Fig 4A, treatment 4). As shown in Fig 4E, progeny virus release is inversely proportional to the ratio of cellular compared to supernatant levels of virus RNA. A lower cell/supernatant ratio corresponds to more progeny virus release. Consequently, the addition of 5 to 40 μg/ml of PA2 was found to significantly inhibit PRRSV virus release from Marc-145 cells in a dose-dependent manner.

### Pre-treatment of PA2 does not affect Marc-145 cell susceptibility to PRRSV

To investigate whether PA2 could also have direct effects on Marc-145 cell susceptibility to PRRSV, serial concentrations of PA2 were added directly to Marc-145 cells for 2 h at 37°C before PRRSV infection (Fig 4A, treatment 5). As shown in Fig 4F, the pre-treatment of PA2 with Marc-145 cells did not reduce viral RNA levels, suggesting that PA2 does not directly affect the susceptibility of Marc-145 cells to PRRSV.

### PA2 treatment reduces cytokine gene expression induced by PRRSV infection in PAMs

**Fig 5:**
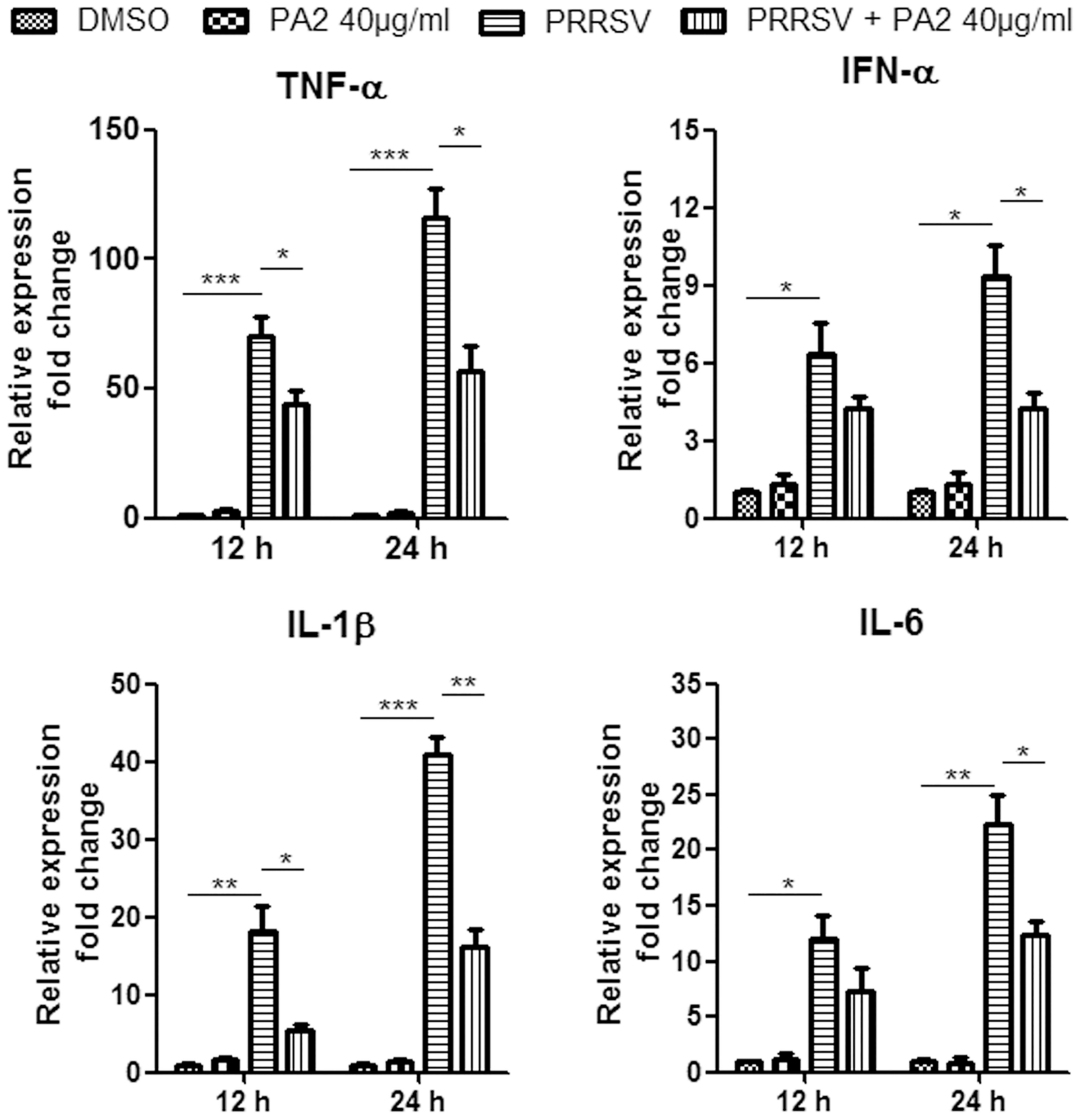
Effect of PA2 treatment on cytokine gene expression induced by PRRSV infection in PAMs. PAMs grown in 6-well plates were infected with PRRSV GD-HD (0.1 MOI) for 2 h at 37°C and then cultured in fresh medium in the presence or absence of 40 μg/ml PA2. Total RNA was extracted from lysates of PAMs at 12 hpi and 24 hpi. The mRNA level of each cytokine gene was assessed by RT-PCR using cellular GAPDH mRNA as the internal loading control. Relative expression (fold change) in comparison with DMSO-treated mock-infected cells (set as 1) is shown. The data represent the mean values from three independent experiments. Statistical significances are denoted by *p < 0.05, **p < 0.01, and ***p < 0.001.

PRRSV infection induces production of cytokines and in return cytokines are able to interfere with viral infection. To investigate whether PA2 treatment affects cytokine production, the expressions of four cytokines including TNF-α, IFN-α, IL-1β and IL-6, known to be involved in antiviral response and inflammation, were analyzed in the presence or absence of PA2. PAMs were incubated with or without 40 μg/ml PA2 for 12 h or 24 h post PRRSV GD-HD infection, and then RT-PCR was performed to assess the relative mRNA level in PAMs. As shown in Fig 5, PRRSV infection significantly induced RNA expressions of the four cytokines while PA2 treatment alone did not alter the expression of cytokines. The expressions of TNF-α exhibited 70- and 110-fold increases at 12 h and 24 hpi, respectively, compared to that of the DMSO control. The expressions of IL-1β and IL-6 exhibited moderate increase, which were less than that of TNF-α but significantly more than that of IFN-α. Notably, PA2 treatment dramatically inhibited PRRSV-induced increase of tested four cytokines in PAMs. The maximum decrease reached 60% in the relative mRNA level of IL-1β at 24 hpi upon PA2 treatment.

## Discussion

In veterinary medicine, vaccination is considered as the golden standard for controlling viral diseases, with very limited usage of anti-viral medications reported for high mutagenicity viruses like PRRSV, however, currently commercially available vaccines have shown limited success in conferring protection against heterologous strains, highlighting a pragmatic need to consider combined therapies with anti-viral agents for effective virus control. Natural products, in particular flavonoids, often harbor many bioactive compounds, which can provide an additional source of novel drug compounds with reported antioxidant, anti-bacterial and anti-viral activities[39–42]. In this study, we showed that PA2 inhibited PRRSV replication in Marc-145 cells at concentrations well below its threshold for cell cytotoxicity. The anti-PRRSV activity of PA2 was confirmed by RT-PCR for decreased NSP9 mRNA levels and by Western blotting for significantly reduced N protein expression. Since Marc-145 cells are not of porcine origin but are monkey cells [43], we also examined the antiviral activity of PA2 in PAMs, which are known to be the primary host target cell for PRRSV infection *in vivo*. Consistent with the data obtained from Marc-145 cells, PA2 strongly inhibited virus replication in PAMs, indicating that it might be an effective inhibitor of PRRSV infection *in vivo*. Our study also used ribavirin, a broad-spectrum anti-viral agent, as a positive control as Amina and co-workers have previously demonstrated ribavirin to be effective in suppressing PRRSV replication in Marc-145 cells [44]. In our study, PA2 exerted similar, if not enhanced anti-viral activity against PRRSV *in vitro*.

Following cell attachment, PRRSV entry into host cells is mediated by receptor-specific endocytosis [38]. We first examined the potential effects of PA2 on PRRSV attachment and found that progeny PRRSV particles were significantly reduced when PA2 was co-incubated with PPRSV infected cells for 2 h at 4°C. We examined whether this effect was mediated by PA2-induced decrease of cell susceptibility to PRRSV, and found that PA2 treatment did not reduce cell susceptibility to PRRSV. These results suggest that the early stage anti-viral effects of PA2 may be mediated directly through PRRSV inactivation or impairment.

We also examined the potential effects of PA2 on virus internalization, viral RNA replication and progeny virus release through PA2 treatment during later time points correlating with PRRSV attachment. We found that PA2 could inhibit virus internalization, as well as progeny virus release. The anti-viral effects of PRRSV, however, did not include the inhibition of viral RNA replication.

Previous studies have demonstrated that the anti-viral mechanisms of proanthocyanidins vary between different virus stains. Laura and co-workers observed that PA2 inhibited Canine Distemper Virus (CDV) *in vitro* by attenuating CDV RNA synthesis and inhibiting progeny virus release during both early and late stages of the viral replication cycle [30]. Gescher and co-workers demonstrated that proanthocyanidin-enriched extracts from *Myrothamnus flabellifolia Welw* exerted antiviral activity against HSV-1 through the inhibition of viral binding and internalization [45]. They observed aggregation of the essential glycoprotein gD from HSV-1 by forming oligomeric structures and speculated that proanthocyanidins might link gD covalently. Similar effects were reported by Fink and co-workers on HIV-1 [46]. Furthermore, Cheng and co-workers showed that proanthocyanidin A-1 (an epimer of PA2) suppressed HSV-2 infection through inhibiting viral attachment and internalization, as well as the inhibition of the later stages of HSV-2 infection [47]. Here, our data demonstrate that PA2 inhibits PRRSV through likely multiple mechanisms including a strong inhibition on viral attachment and internalization and a moderate inhibition on progeny virion release, in a similar manner to the effects of proanthocyanidin against HSV-1, HSV-2 and HIV-1 infections. Therefore, it could be speculated that proanthocyanidins in general might be considered as entry blockers for enveloped viruses, including PRRSV, through altering the structures of viral envelop proteins.

Interestingly, Xu and others showed that PA2 exhibited significant antioxidant activities *in vitro* [48, 49]. On the other hand, PRRSV infection has been shown to induce oxidative stress in cells by generating reactive oxygen species (ROS) which contributed to the activation of the NF-κB pathway, a biologically significant aspect of PRRSV pathogenesis [50]. Therefore, we could infer that the inhibitory activity of PA2 against PRRSV infection could partly be related to its potent antioxidant activity although this needs to be further investigated.

PRRS virus replicates preferentially in cells of the macrophage lineage, which play a major role in the inflammatory and immune responses [51, 52]. The pro-inflammatory cytokines, TNF-α, IL-1β and IL-6, are among the first cytokines to be produced by the alveolar macrophages during PRRSV infection. Increased levels of these cytokines in the circulation are responsible for the signs of acute systemic inflammation, including fever, depression and anorexia [53]. Inversely, the production of type I interferons (IFNs) IFN-α and IFN-β by virus-infected cells is the most effective innate anti-viral immune response [54]. A significant amount of research has demonstrated that PRRSV has evolved to inhibit the IFN-α respond for evading host immune surveillance [55]. Here we confirmed that PRRSV infection induced significant RNA expression of three pro-inflammatory cytokines TNF-α, IL-1β and IL-6, but a relative meager increase of IFN-α RNA expression. PA2 treatment dramatically inhibited PRRSV-induced increase of RNA expression of TNF-α, IL-1β and IL-6 in PAMs, while for mock-infected PAMs PA2 treatment did not alter the expression of cytokines. Anti-inflammatory effects of proanthocyanidin had been intensively investigated by Ahmad and other researchers. Ahmad and co-workers showed that grape seed proanthocyanidin extract protected mice against carrageenan-induced lung inflammation through the down-regulation of pro-inflammatory cytokines (TNF-α, IL-1β, IL-6 and IFN-γ) and chemokine MCP-1 and inhibition of infiltration of inflammatory cells to the damaged area [56]. Jiang and co-workers reported that proanthocyanidin prevented lipopolysaccharide-induced depressive-like behavior in mice via neuroinflammatory pathway. They further demonstrated that proanthocyanidin inhibited the LPS-induced iNOS and COX-2 overexpression via modulating NF-κB pathway and thus impaired overexpression of pro-inflammatory cytokines (TNF-α, IL-1β and IL-6) in the hippocampus [57]. Therefore, it could be speculated that PA2 could directly intervene cellular immune response and therefore inhibit the expression of pro-inflammatory cytokines induced by PRRSV infection. It should be noted that the decrease of pro-inflammatory cytokines is partly attributed to inhibition of PA2 on virus prolification by which indirectly reduces the expression of pro-inflammatory cytokines.

In conclusion, our study has demonstrated that PA2 is an inhibitor of PPRSV infections *in vitro*, and targets multiple stages of PPRSV infection. This may be particularly advantageous for usage as an anti-viral agent, especially considering that proanthocyanidine is abundantly found in fruits, nuts and seeds. Further *in vivo* studies will be necessary to confirm whether PA2 may act as a novel and effective inhibitor of PPRSV infections in swine.

## Supporting information

S1 Fig A micrograph of PAMs from bronch alveolar lung fluid of piglets at 2h post incubation. For obtaining porcine alveolar macrophages (PAMs), the lungs and heart were removed from 4- to 6-week-old PRRSV-negative Large-White piglets. The lungs were washed three times with pre-cooled phosphate buffered saline (PBS) solution containing penicillin (300 IU/ml) and streptomycin (300 μg/ml). Cells were centrifuged at 800 g for 10 minutes and resuspended in RPMI 1640 supplemented with 10% FBS and 100 IU/ml of penicillin and 100 μg/ml streptomycin at 1× 10^6^ cells/ml in 6-well plate, and then incubated at 37°C for 2 h. The suspending cells (mainly lymphocytes and red blood cells) were removed and adherent cells were PAMs.
(TIF)

S2 Fig HPLC-MS/MS chromatography (A) and mass spectra (B and C) of PA2 from DMEM medium. Analyses were carried out using an Agilent 1200 series HPLC system coupled with an Applied Biosystem API 4000 triple quadrupole mass spectrometer. Chromatographic separation was performed using an Agilent Zorbax SB-Aq C18 column (150 mm × 2.1 mm i.d., 3.5μm). The mass analysis was carried out under the negative electrospray ionization mode. The transitions of m/z 575.2→288.8 was used for quantification. (A) Total ions chromatogram of PA2. (B) ESI(-) full scan mass spectra of PA2. (C) The secondary mass spectra of PA2 (m/z 575.2).
(TIF)

S3 Fig Dynamic virus titer in Marc-145 cell cultures. Marc-145 cells grown in 6-well plates were infected with PRRSV GD-XH (0.05 MOI) for 2 h at 37°C and then cultured in fresh medium. At indicated time-points post infection, the samples (mixture of supernatants and cells) were subjected to viral titer titration using the end point dilution assay and expressed as log_10_ TCID_50_/ml.
(TIF)

S4 Fig The anti-PRRSV activity of PA2 in PAM cultures. PAMs grown in 6-well plates were infected with PRRSV GD-HD or GD-XH or CH-1a (0.1 MOI) for 2 h at 37°C and then cultured in fresh medium containing various concentrations of PA2. Relative PRRSV NSP9 mRNA expression of PA2 treated groups to DMSO-treated control (0 μg/ml PA2) (set as 1) was analyzed using real-time RT-PCR at 24 h after treatment with PA2. Data are the mean values from three independent experiments. *p < 0.05, **p < 0.01, and ***p < 0.001 compared to DMSO-treated control.
(TIF)

S5 Fig PA2 blocks attachment and internalization of PRRSV. MARC-145 cells were infected with the PRRSV GD-XH strain at 0.5 MOI. The infected cells were cultured in the presence of a series of concentrations of PA2 and collected at indicated time-points post infection for determination of the relative expression level of viral NSP9 mRNA to DMSO-treated control (0 μg/ml PA2) by qRT-PCR. (A) Viral binding assay. Marc-145 cells were incubated with essential medium containing PRRSV in the presence of PA2 or a control at 4°C for 2 h to facilitate virus binding. Cells were then washed three times with PBS to remove any unbound virus particles and chemicals and then submitted to real-time PCR analysis; (B) Viral internalization assay. Marc-145 cells were incubated with essential medium containing PRRSV at 4°C for 2 h. After three washes with PBS, cells were placed in fresh medium and cultured at 37°C to facilitate virus internalization. A serial dilutions of PA2 were then added for 3 h treatment. Cells were then washed three times with PBS to remove free virus particles and chemicals and submitted to real-time PCR analysis.
(TIF)
