# Supplementary figures and images for: Inhibition of proanthocyanidin A2 on porcine reproductive and respiratory syndrome virus replication in vitro
Source: PLoS One. 2018 Feb 28;13(2):e0193309. doi: 10.1371/journal.pone.0193309 (PMC5831109; doi:10.1371/journal.pone.0193309)

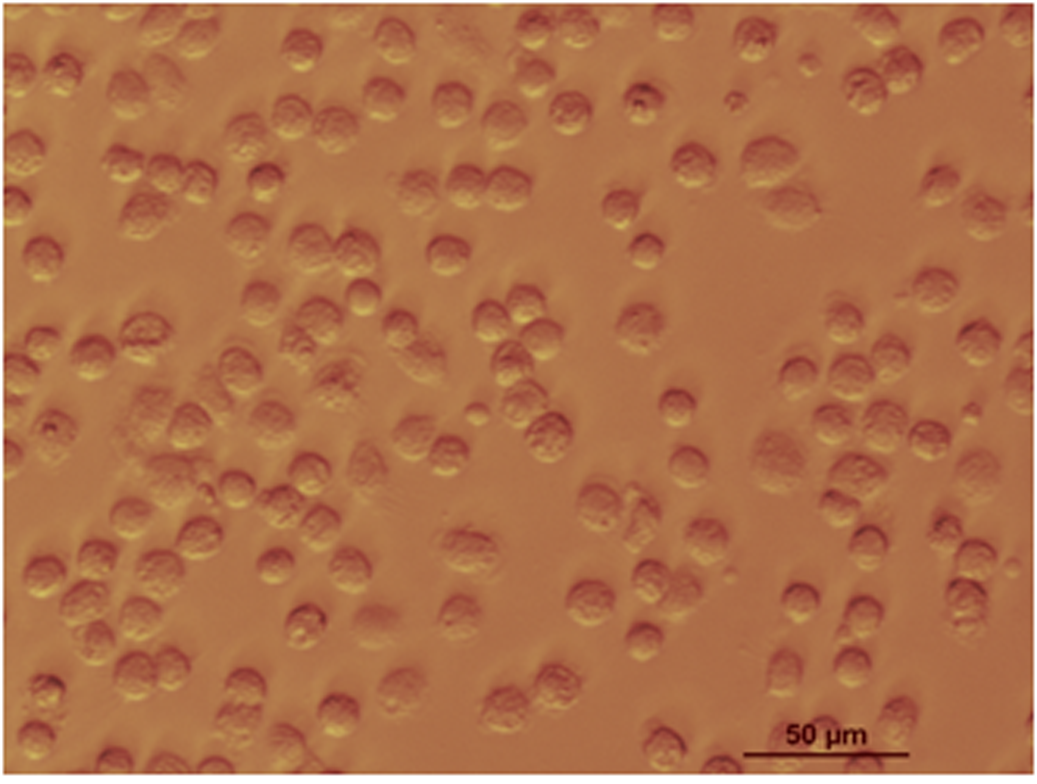

Supplement: S1 Fig — For obtaining porcine alveolar macrophages (PAMs), the lungs and heart were removed from 4- to 6-week-old PRRSV-negative Large-White piglets. The lungs were washed three times with pre-cooled phosphate buffered saline (PBS) solution containing penicillin (300 IU/ml) and streptomycin (300 μg/ml). Cells were centrifuged at 800 g for 10 minutes and resuspended in RPMI 1640 supplemented with 10% FBS and 100 IU/ml of penicillin and 100 μg/ml streptomycin at 1× 10^6^ cells/ml in 6-well plate, and then incubated at 37°C for 2 h. The suspending cells (mainly lymphocytes and red blood cells) were removed and adherent cells were PAMs. (TIF) [file pone.0193309.s001.tif]

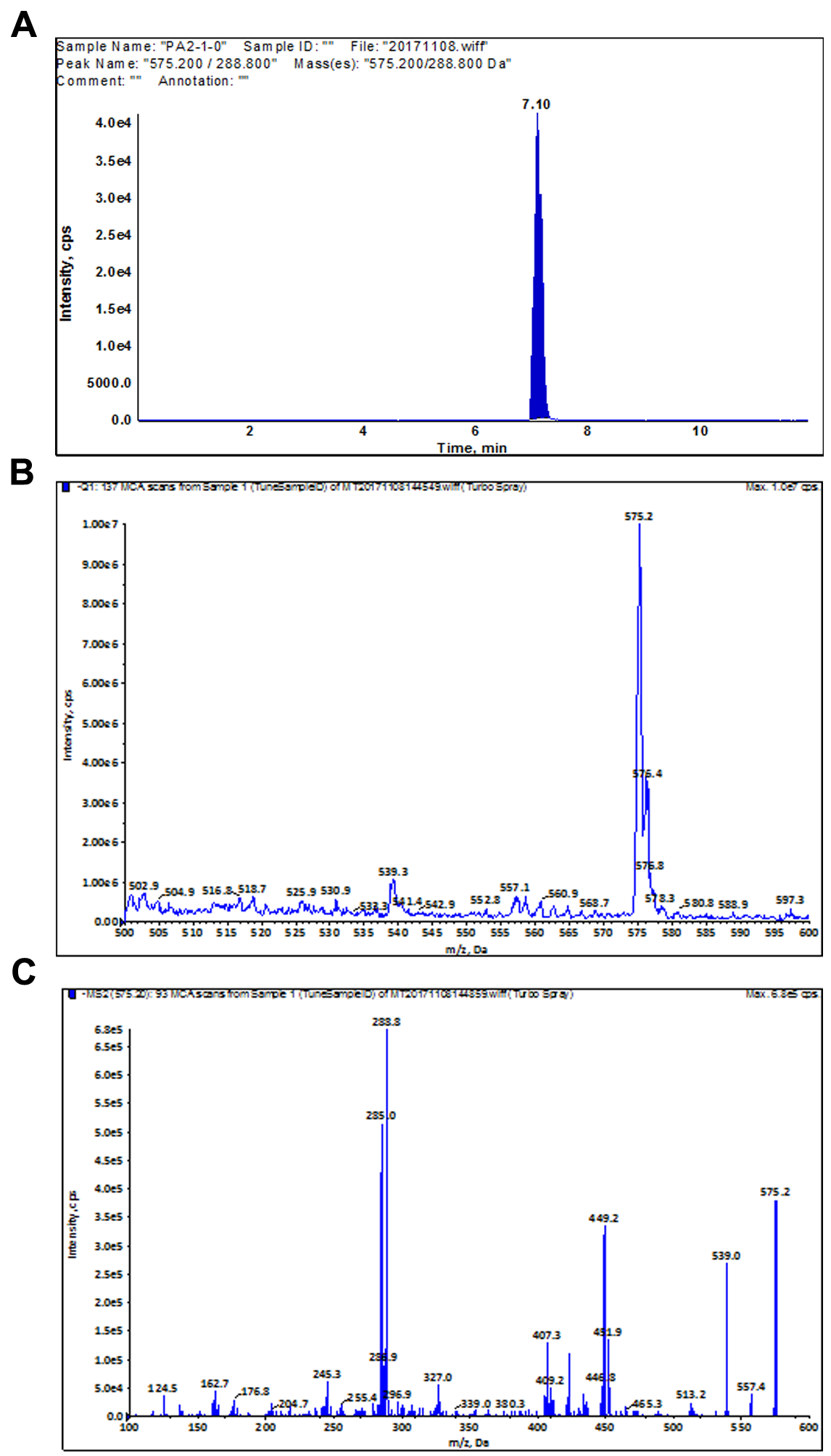

Supplement: S2 Fig — Analyses were carried out using an Agilent 1200 series HPLC system coupled with an Applied Biosystem API 4000 triple quadrupole mass spectrometer. Chromatographic separation was performed using an Agilent Zorbax SB-Aq C18 column (150 mm × 2.1 mm i.d., 3.5μm). The mass analysis was carried out under the negative electrospray ionization mode. The transitions of m/z 575.2→288.8 was used for quantification. (A) Total ions chromatogram of PA2. (B) ESI(-) full scan mass spectra of PA2. (C) The secondary mass spectra of PA2 (m/z 575.2). (TIF) [file pone.0193309.s002.tif]

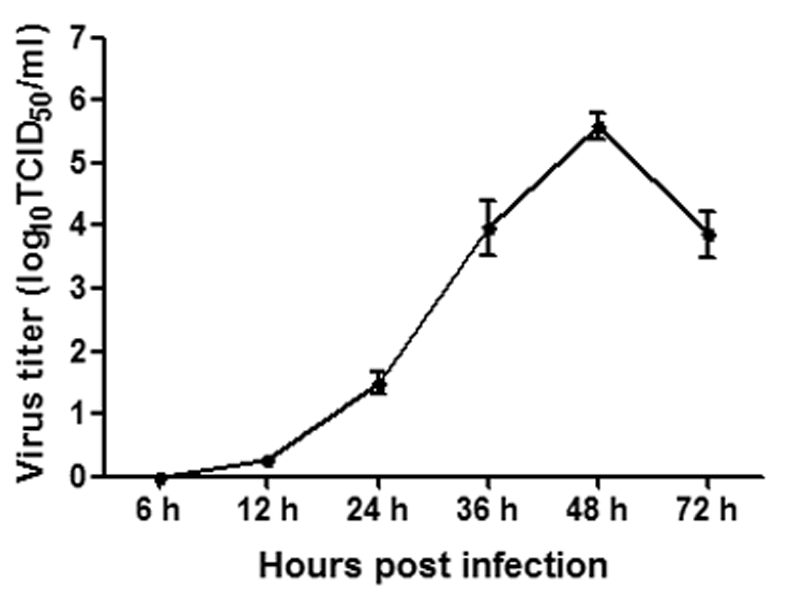

Supplement: S3 Fig — Marc-145 cells grown in 6-well plates were infected with PRRSV GD-XH (0.05 MOI) for 2 h at 37°C and then cultured in fresh medium. At indicated time-points post infection, the samples (mixture of supernatants and cells) were subjected to viral titer titration using the end point dilution assay and expressed as log10 TCID50/ml. (TIF) [file pone.0193309.s003.tif]

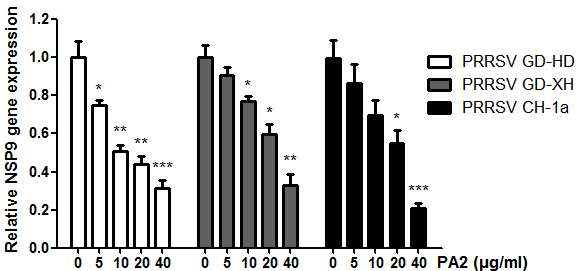

Supplement: S4 Fig — PAMs grown in 6-well plates were infected with PRRSV GD-HD or GD-XH or CH-1a (0.1 MOI) for 2 h at 37°C and then cultured in fresh medium containing various concentrations of PA2. Relative PRRSV NSP9 mRNA expression of PA2 treated groups to DMSO-treated control (0 μg/ml PA2) (set as 1) was analyzed using real-time RT-PCR at 24 h after treatment with PA2. Data are the mean values from three independent experiments. *p < 0.05, **p < 0.01, and ***p < 0.001 compared to DMSO-treated control. (TIF) [file pone.0193309.s004.tif]

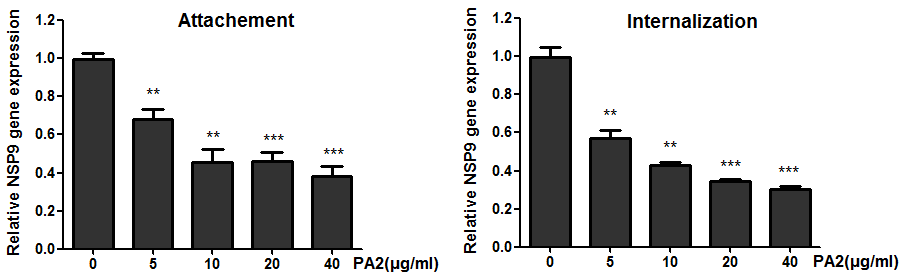

Supplement: S5 Fig — MARC-145 cells were infected with the PRRSV GD-XH strain at 0.5 MOI. The infected cells were cultured in the presence of a series of concentrations of PA2 and collected at indicated time-points post infection for determination of the relative expression level of viral NSP9 mRNA to DMSO-treated control (0 μg/ml PA2) by qRT-PCR. (A) Viral binding assay. Marc-145 cells were incubated with essential medium containing PRRSV in the presence of PA2 or a control at 4°C for 2 h to facilitate virus binding. Cells were then washed three times with PBS to remove any unbound virus particles and chemicals and then submitted to real-time PCR analysis; (B) Viral internalization assay. Marc-145 cells were incubated with essential medium containing PRRSV at 4°C for 2 h. After three washes with PBS, cells were placed in fresh medium and cultured at 37°C to facilitate virus internalization. A serial dilutions of PA2 were then added for 3 h treatment. Cells were then washed three times with PBS to remove free virus particles and chemicals and submitted to real-time PCR analysis. (TIF) [file pone.0193309.s005.tif]
